# Open-Label Placebo Injection for Chronic Back Pain With Functional Neuroimaging: A Randomized Clinical Trial

**DOI:** 10.1001/jamanetworkopen.2024.32427

**Published:** 2024-09-11

**Authors:** Yoni K. Ashar, Michael Sun, Karen Knight, Thomas F. Flood, Zachary Anderson, Ted J. Kaptchuk, Tor D. Wager

**Affiliations:** 1 Division of General Internal Medicine, University of Colorado Anschutz Medical Campus, Aurora; 2 Department of Psychological and Brain Sciences, Dartmouth College, Hanover, New Hampshire; 3 Panorama Orthopedics and Spine Center, Golden, Colorado; 4 Department of Radiology, Brigham and Women’s Hospital, Boston, Massachusetts; 5 Department of Psychology, Northwestern University, Evanston, Illinois; 6 Beth Israel Deaconess Medical Center, Harvard Medical School, Boston, Massachusetts

## Abstract

**Importance:**

Chronic back pain (CBP) is a leading cause of disability. Placebo treatments often provide as much pain relief as bona fide treatments, such as steroid injections. Open-label (honestly prescribed) placebos (OLPs) may relieve CBP without deception, but OLP mechanisms remain poorly understood.

**Objective:**

To investigate the long-term efficacy and neurobiological mechanisms of OLP for CBP.

**Design, Setting, and Participants:**

A randomized clinical trial of CBP with longitudinal functional magnetic resonance imaging (MRI) comparing OLP with usual care, with 1-year follow-up, was conducted in a university research setting and a community orthopedic clinic. Participants were individuals aged 21 to 70 years with CBP. The trial was conducted from November 2017 to August 2018, with 1-year follow-up completed by November 2019. Data analysis was performed from April 2020 to May 2024. The primary analysis was conducted on an intention-to-treat sample.

**Interventions:**

Participants randomized to OLP received a 1-time subcutaneous lumbar saline injection presented as placebo accompanied by information about the power of placebo to relieve pain, alongside their ongoing care. Usual care participants continued their ongoing care.

**Main Outcomes and Measures:**

The primary outcome was pain intensity (0-10, with 0 indicating no pain and 10 the most intense) at 1 month posttreatment. Secondary outcomes included pain interference, depression, anxiety, anger, and sleep quality. Functional MRI was performed before and after treatment during evoked and spontaneous back pain.

**Results:**

A total of 101 adults (52 [51.4%] females; mean [SD] age, 40.4 [15.4] years) with moderate severity CBP (mean [SD], 4.10 [1.25] intensity; duration, 9.7 [8.5] years) were enrolled. Compared with usual care, OLP reduced CBP intensity posttreatment (relative reduction, 0.61; Hedges *g* = 0.45; 95% CI, −0.89 to 0.04; *P* = .02). Through 1-year follow-up, pain relief did not persist, although significant benefits were observed for depression, anger, anxiety, and sleep disruption (Hedges *g* = 0.3-0.5; all *P* < .03). Brain responses to evoked back pain for OLP vs usual care increased in rostral anterior cingulate and ventromedial prefrontal cortex and decreased in somatomotor cortices and thalamus. During spontaneous pain, functional connectivity analyses identified OLP vs usual care increases in ventromedial prefrontal cortex connectivity to the rostral ventral medulla, a pain-modulatory brainstem nucleus. No adverse effects of treatment were reported by participants.

**Conclusions and Relevance:**

In this randomized clinical trial of OLP vs usual care, a single nondeceptive placebo injection reduced CBP intensity for 1 month posttreatment and provided benefits lasting for at least 1 year posttreatment. Brain mechanisms of OLP in a clinical population overlap with those of deceptive placebos in healthy volunteers, including engagement of prefrontal-brainstem pain modulatory pathways.

**Trial Registration:**

ClinicalTrials.gov Identifier: NCT03294148

## Key Points

**Question:**

What are the clinical effects and brain mechanisms of open-label (honestly prescribed) placebos for chronic back pain?

**Findings:**

In this randomized clinical trial of 101 adults with chronic back pain, an open-label subcutaneous placebo (saline) injection led to significant improvements in pain intensity, mood, and sleep at 1 month posttreatment compared with usual care. The placebo treatment also led to reduced somatomotor activity and increased medial prefrontal activity during evoked back pain and to increased medial prefrontal-brainstem functional connectivity during spontaneous pain.

**Meaning:**

The findings of this trial suggest that open-label placebo treatments can confer meaningful clinical benefits to patients with chronic back pain by engaging prefrontal-brainstem pathways linked to pain regulation and opioidergic function.

## Teaser

This randomized clinical trial examines the use of open-label placebo injections in individuals with chronic back pain.

## Introduction

Placebo or sham treatments for chronic pain are powerful: in many cases, they provide as much or nearly as much pain relief as bona fide pills, injections, and surgeries.^1,2,3,4^ Traditionally, the efficacy of placebo treatment was thought to hinge on deception of the patient, creating the illusion of an active treatment being administered. Yet, research has upended this belief by investigating open-label placebo (OLP) treatments, which are disclosed to both patients and clinicians as placebo.^5^

Open-label placebo treatments have demonstrated benefits for several conditions, including migraine, cancer-related fatigue, irritable bowel syndrome, and chronic back pain (CBP).^6,7,8,9^ Chronic back pain is a leading cause of disability globally and a top contributor to medical expenditures in the US.^10,11,12^ In most cases, peripheral pathologic factors (eg, disc bulge) cannot explain CBP, and plasticity in central nervous system processes is the predominant cause of ongoing pain.^13,14,15^ Open-label placebo treatments, which primarily engage brain and behavioral processes, may thus target core mechanisms of CBP. Two prior trials have demonstrated that OLP treatments can reduce CBP intensity,^16,17^ but it remains unknown how OLP treatments engage putative brain mechanisms to relieve CBP.

Placebo neuroimaging studies have focused on traditional (deceptive) placebo treatments in healthy volunteers in experimental pain paradigms (eg, heat pain applied to the forearm). Broadly, these studies have identified 3 major findings induced by placebo manipulations: decreased activity in brain regions related to somatosensory and nociceptive processing (eg, thalamus, somatomotor cortex), increased activity in prefrontal pain-regulatory regions (eg, rostral anterior cingulate cortex [rACC], ventromedial prefrontal cortex [vmPFC], dorsolateral prefrontal cortex [dlPFC]), and the engagement of multiple brainstem nuclei modulating afferent input and exerting descending control, especially the periaqueductal gray (PAG) and rostral ventral medulla (RVM).^18,19,20,21,22,23,24,25^ Yet, how the brain mechanisms identified in laboratory paradigms testing healthy volunteers compare with those of patients receiving clinical treatments remains poorly understood.^26,27^ To our knowledge, the brain mechanisms of an OLP treatment in a patient population have never been investigated.

In the present study, we sought to evaluate the effects of a novel OLP treatment—a 1-time subcutaneous injection of saline into the back. We measured multiple patient-reported outcomes during a 1-year follow-up period, as prior studies have provided conflicting evidence on the durability of OLP effects in CBP.^28,29^ We conducted longitudinal functional MRI (fMRI) to assess the effects of OLP on back pain–related brain activity and on functional connectivity during spontaneous pain. We hypothesized that the neurobiological effects of OLP in CBP would resemble the neuroimaging findings from laboratory pain paradigms.

## Methods

The trial was conducted from November 2017 to August 2018, with a 1-year follow-up completed by November 2019. The trial was designed to facilitate 2 comparisons of interest: a test of a psychotherapy intervention, with OLP serving as a control condition described earlier,^30^ and the comparison of OLP vs usual care on mechanistic and clinical outcomes—the focus of this article; the protocol is reported in Supplement 1. Participants provided written informed consent as approved by the University of Colorado Institutional Review Board and received financial compensation. Our report follows the Consolidated Standards of Reporting Trials (CONSORT) reporting guideline.

### Participants

Participants were recruited from the community using electronic and print announcements, social media, and referrals in 2017-2018. Recruitment materials described a mind-body treatment for CBP, explained to be an honest placebo during informed consent.

Participants aged 21 to 70 years with back pain for at least half the days of the past 6 months and 1-week average pain intensity of 4 or greater on a 10-point scale (0, no pain; 10, the most intense pain) at screening were recruited from the Boulder, Colorado, area. We targeted primary CBP, excluding patients with leg pain worse than back pain and self-reported diagnoses of inflammatory disorders or metastasizing cancers. We excluded people self-reporting psychosis, personality disorders, pain-related compensation or litigation in the past year, or inability to undergo MRI (details provided in the eMethods in Supplement 2). Power analysis targeted 80% power (α = .05) to detect a medium effect (*d* = 0.62) on pain intensity at the primary end point (eMethods in Supplement 2). Participants self-reported race and ethnicity to characterize the sample, per recommendations.

Participants completed an eligibility/consent session and a baseline assessment session with fMRI. They were subsequently randomized using an imbalance-minimization algorithm^31^ to OLP or usual care, balancing on age, sex, baseline pain, and opioid use (eMethods in Supplement 2). Participants were not blinded due to the nature of the intervention. All research staff collecting data were blinded to group assignment.

**Figure 1.**
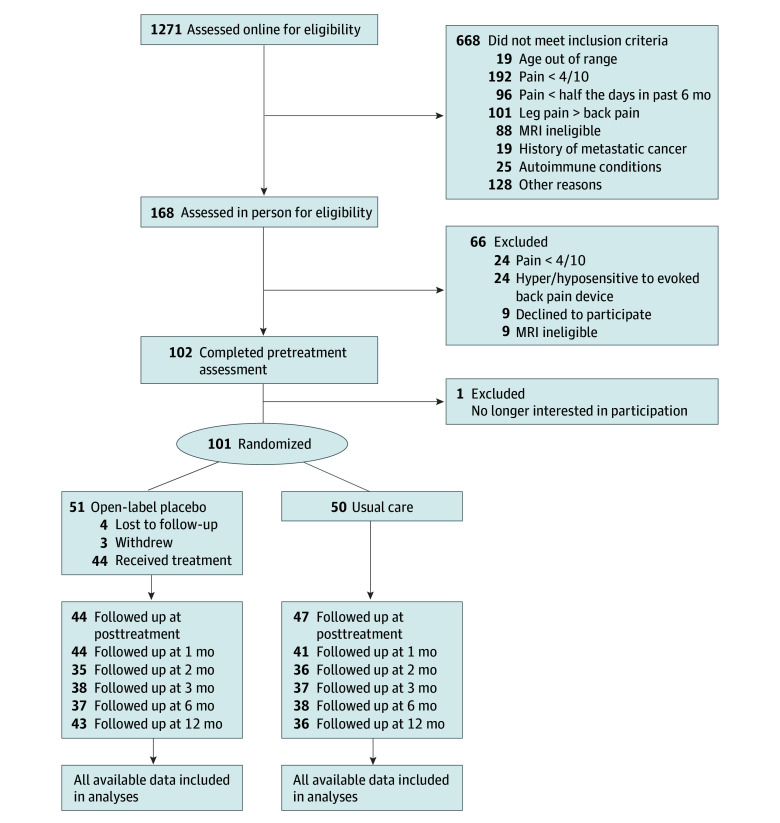
Participant Flow Through the Trial MRI indicates magnetic resonance imaging.

The primary end point (posttreatment fMRI session) occurred 1 month after the baseline fMRI session. Participants completed online follow-up assessments at 1, 2, 3, 6, and 12 months after the posttreatment session (Figure 1). Adverse events were recorded when participants spontaneously reported them to study personnel.

Half the participants in the usual care arm were from a parallel, simultaneous clinical trial testing a psychotherapeutic intervention vs usual care. To increase statistical power, we designed these 2 trials to support combining the 2 usual care arms: both trials recruited from an identical population using identical recruitment methods, collected identical assessment measures, and had the same instructions for the usual care arm.

### Interventions, Materials, and Procedures

#### Open-Label Placebo

Open-label placebo included an integrated cognitive, social, and physical (injection) intervention. Participants presented to a private orthopedic medical center in Golden, Colorado. They watched 2 videos (available for reuse on request) and had a structured conversation with the treating physician (K.K.) in the context of an empathic, validating clinical encounter. The videos and conversation aimed to convey that (1) they were receiving a placebo—an inert treatment with no active ingredients; (2) placebos can have powerful effects; (3) placebos produce endogenous opioid release, establishing a rationale for pain relief; (4) placebos can work even when known to be inert by engaging automatic/nonconscious pathways (eg, automatically triggering the body’s natural healing response); and (5) a positive attitude may be helpful but is not necessary, encouraging instead an open-minded attitude.^32^ Participants changed into a medical gown, and a subcutaneous injection described as saline with no active medication was administered at the site of the greatest back pain. Participants also continued any ongoing usual care for their back pain and agreed not to begin new treatments.

#### Usual Care

The usual care participants were given no additional treatment by the study staff. They agreed to continue their ongoing care as usual and not start new treatments.

### Clinical Measures

#### Clinical Outcomes

The primary outcome was average pain over the last week on an 11-point numeric rating scale (0, no pain; 10, worst pain imaginable), as assessed with the Brief Pain Inventory–Short Form (BPI-SF).^33^ We adopted this as the primary outcome owing to its enhanced interpretability, high correlations (*r* > .90) with the full BPI-SF severity scale scores, and recommendations from a National Institutes of Health task force and the scale developers.^33,34,35^ Secondary outcomes included pain interference (BPI-SF); Patient-Reported Outcomes Measurement Information System short forms for depression, anxiety, anger, and sleep quality^36,37^; Patient Global Impression of Change, and the Treatment Satisfaction Questionnaire^38^ (eMethods in Supplement 2 provides measure details). Outcomes were collected at prerandomization and at all follow-up time points, except the Patient Global Impression of Change and Treatment Satisfaction Questionnaire, which cannot be measured before randomization. Baseline values for primary and secondary outcomes were computed as the mean score from 2 prerandomization assessments (eligibility session and pretreatment fMRI session). Additional measures of psychological functioning were obtained at baseline for testing as potential moderators of OLP response (eMethods in Supplement 2).

### Neuroimaging Measures

We acquired both structural (T1 magnetization–prepared rapid gradient echo imaging) and functional images (multiband echo planar imaging). Sequence parameters and a complete description of neuroimaging methods are provided in the eMethods in Supplement 2.

#### Evoked Back Pain

During fMRI, participants completed an evoked back pain task with a series of randomly ordered trials distending the back to 1 of 4 intensity levels. The evoked back pain task used a novel device providing experimental control over back pain during fMRI. Participants lay on a pneumatically controlled cylindrical balloon, with increasing inflation causing increasingly painful back distention, and rated pain after each trial on a visual analog scale (0 indicates no pain; 100, worst pain imaginable).

#### Spontaneous Pain (Resting State)

An 8-minute scan was performed for each participant before and after treatment. Participants were asked to keep their eyes open and fixate on a visual crosshair; once per minute, participants rated their spontaneous back pain intensity on a visual analog scale.

### Statistical Analysis

#### Clinical Outcomes

Intention-to-treat analyses including all randomized patients were performed. Primary and secondary outcome scores were modeled at posttreatment (the primary end point) with a mixed-effects model (fitlme, MATLAB 2023a) at a *P* < .05 significance level. Regressors included dummy-coded treatment group (OLP vs usual care) and time point (post vs pre) variables, a group × time interaction (OLP vs usual care × post vs pre), covariates for age and sex, and a random intercept and slope per participant. Treatment response rates were computed as the percentage of randomized participants reporting 30% or more and 50% or more pain reduction posttreatment.

Effects of OLP on primary and secondary outcomes at 1, 2, 3, 6, and 12 months posttreatment were examined in 3 ways. First, we tested for OLP effects throughout the entire follow-up period in models including data from all follow-up time points. Regressors included a dummy-coded treatment group variable, a time point variable indicating months posttreatment and mean centered at 6 months (the midpoint of the 12-month follow-up period), a group × time interaction, covariates for age and sex, and a random intercept and slope per participant. Time was centered at 6 months posttreatment to maximize power for detecting group effects throughout the entire follow-up period. Estimated effects of group can be interpreted as group differences at 6 months posttreatment, with the group × time interaction testing for changes in OLP vs usual care effects across the 12-month follow-up period. Second, we estimated OLP vs usual care effect sizes (Hedges *g*) at each follow-up time point for each outcome, adjusting for baseline values of the outcome (eMethods in Supplement 2). Third, we tested whether these OLP vs usual care effect sizes were significant at 12 months posttreatment—our longest follow-up time point.

Self-reported pain during the evoked back pain task (mean pain across trials) was also submitted to a mixed-effects model, as described in the first paragraph of this section, testing for a group × time interaction effect. We further conducted exploratory analyses testing baseline measures of psychological functioning as predictors of response to OLP (eMethods in Supplement 2).

### Neuroimaging Analyses

#### Preprocessing and Denoising

Standard fMRI preprocessing procedures were used, implemented in fMRIPrep 1.2.4^39^ which is based on Nipype 1.1.6.^40^ This included coregistration, normalization of anatomic images to a template image (ICBM 152 Nonlinear Asymmetric template version 2009c), susceptibility artifact distortion correction, and smoothing with a 6-mm kernel.

#### Evoked Pain Task

A first-level model was estimated for each participant to identify brain activity associated with evoked back pain intensity. We constructed a continuous within-person estimate of evoked pain intensity based on posttrial pain ratings. This modeled pain experience throughout the evoked back pain task and provided a contrast image for each participant, estimating how strongly each voxel was related to evoked pain (eMethods in Supplement 2). Multiple covariates in the first-level model controlled for head motion effects (eMethods in Supplement 2).

Second-level models tested for OLP vs usual care effects on evoked back pain–related brain activity. We conducted a voxelwise robust regression using SPM12 and the CanlabCore toolbox^41^ to estimate the OLP vs usual care effect at posttreatment, controlling for age, sex, and pretreatment values at the given voxel.^42,43^

Statistical thresholding was conducted using a nonparametric combination testing framework correcting both within and across regions of interest (ROIs).^44^ We defined 6 ROIs reliably associated with placebo analgesia in prior meta-analyses,^18,20^ including 2 areas showing placebo-induced increases (vmPFC/rACC, dlPFC) and 4 areas showing placebo-induced decreases (insula, midcingulate, medial somatomotor cortex, thalamus) (eMethods, eFigure 1 in Supplement 2). A permutation test conducted within each ROI was thresholded at *P* < .05 familywise error rate (FWER) corrected across voxels, along with a permutation-based correction across ROIs (FWER *P* < .05 across the set of ROIs) (eMethods in Supplement 2).^44^ Whole-brain uncorrected results are reported for archival purposes (eMethods, eTable 2 in Supplement 2).

#### Connectivity Analyses

Two vmPFC regions identified in evoked pain analyses were submitted as seed regions to test for placebo-induced increases in spontaneous (resting) connectivity with the PAG and RVM, as shown in placebo analgesia studies,^24,25,45^ with nonparametric combination testing to correct for multiple comparisons (eMethods in Supplement 2). The spontaneous pain (resting state) task was preprocessed as above, along with global signal regression and bandpass filtering (0.1-0.01 Hz) (eMethods in Supplement 2). Periaqueductal gray and RVM were defined anatomically using a high-resolution brainstem atlas.^46^

## Results

A total of 101 participants were randomized. The sample included 52 females (51.4%) and 49 males (48.6%), with mean (SD) age, 40.4 (15.4) years, and with all participants reporting at least some college education (Table 1). Of the 101 participants, 1 (1.0%) was American Indian or Alaska Native, 2 (2.0%) were Asian/Pacific Islander, 3 (3.0%) were Black, 88 (87.1%) were White, and 7 (7.0%) were other or unknown, with 4 (4.0%) participants of Hispanic ethnicity (Table 1). The sample had moderate pain intensity (mean [SD], 4.10 [1.25]) at pretreatment, with mean (SD) CBP duration of 9.7 (8.5) years. Ninety-one individuals (90.1%) completed the posttreatment assessment session (Figure 1). Of 51 participants randomized to OLP, 4 (7.8%) were lost to follow-up and 3 (5.8%) withdrew from treatment (Figure 1). Of 50 participants randomized to usual care, 3 (6.0%) did not complete the posttreatment assessment (Figure 1).

**Table 1.** Participant Demographic Characteristics

Characteristic	No. of patients (%)	
	OLP	Usual care
Age, mean (SD), y	39.4 (14.9)	41.3 (15.9)
Sex		
Female	25 (49.0)	27 (54.0)
Male	26 (50.9)	23 (46.0)
Education		
High school or less	0	0
Some college	15 (29.4)	15 (30.0)
College graduate	36 (70.6)	35 (70.0)
Married	25 (49.0)	30 (60.0)
Race and ethnicity^a^		
American Indian or Alaska Native	0	1 (2.0)
Asian/Pacific Islander	2 (3.9)	0
Black (not of Hispanic origin)	2 (3.9)	1 (2.0)
Hispanic ethnicity	2 (3.9)	2 (4.0)
White (not of Hispanic origin)	45 (88.2)	43 (86.0)
Other or unknown^b^	2 (3.9)	5 (10.0)
Employment status		
Full time (>30 h/wk)	26 (51.0)	28 (56.0)
Part time (5-30 h/wk)	12 (23.5)	13 (26.0)
Unemployed/lightly employed (<5 h/week)	13 (25.5)	9 (18.0)
SSES mean (SD), 1-10	6.4 (2.0)	6.7 (1.6)
Exercise, h/wk		
Almost none	1 (2.0)	4 (8.0)
1	7 (13.7)	9 (18.0)
3	23 (45.1)	14 (28.0)
7	18 (35.3)	21 (42.0)
≥14	2 (3.9)	2 (4.0)
Pain-related characteristics		
Pain duration, mean (SD), y	8.9 (8.2)	10.5 (8.9)
Current opioid use	2 (3.9)	2 (4.0)
Pain in body sites besides back		
None	9 (17.6)	4 (8.0)
A little	24 (47.1)	28 (56.0)
A moderate amount	15 (29.4)	16 (32.0)
A lot	3 (5.9)	2 (4.0)

Abbreviation: SSES, subjective socioeconomic status, rated on a 1 to 10 ladder.

^a^ Participants self-reported race and ethnicity, which was reported to characterize the sample, per recommendations.

^b^ No further breakdown of this classification is available.

### Patient-Reported Outcomes

**Figure 2.**
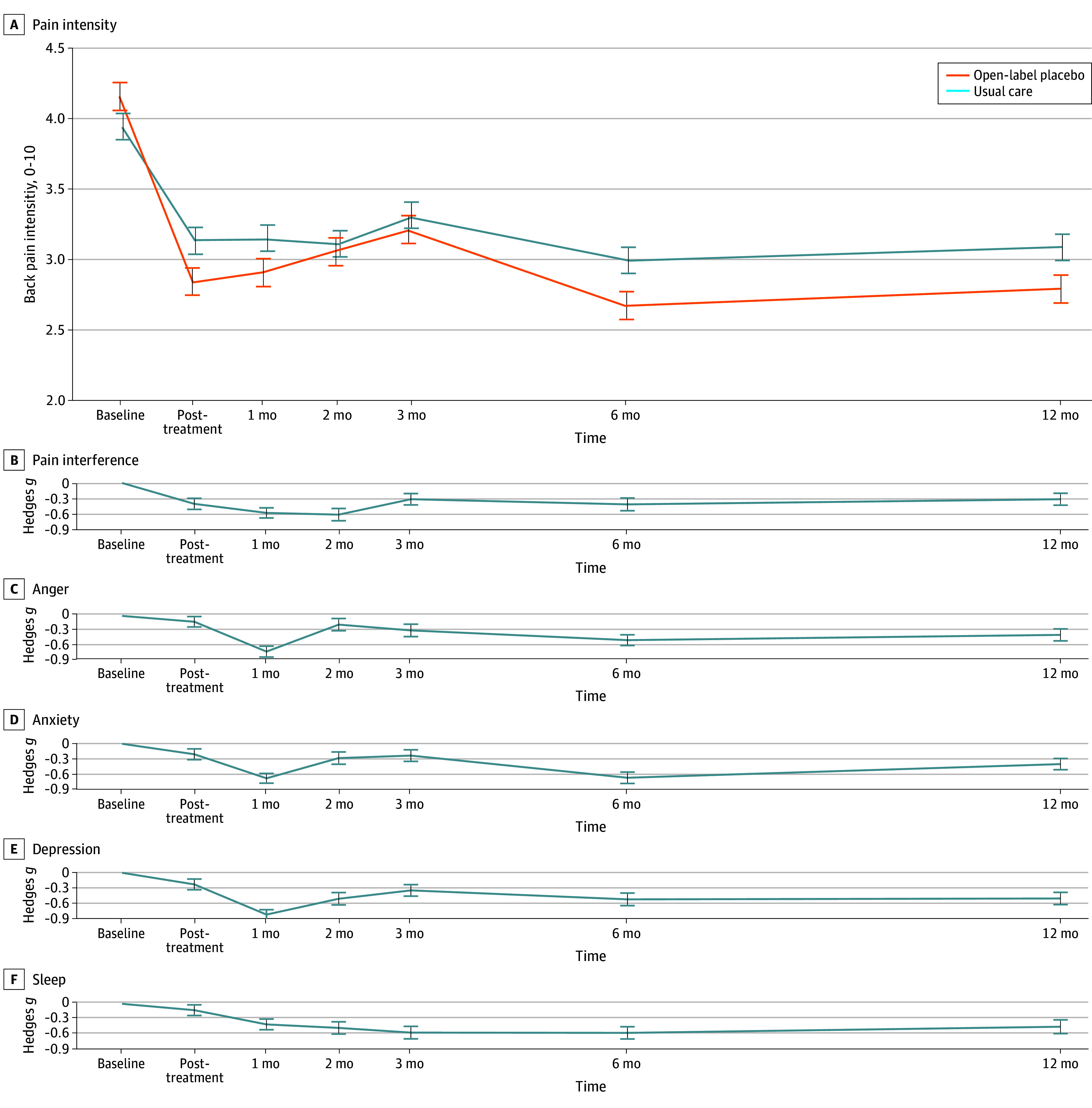
Effects of Open-Label Placebo (OLP) vs Usual Care on Patient-Reported Outcomes Through 1-Year Follow-Up A, OLP vs usual care led to reduced chronic back pain intensity (primary outcome, 0-10 scale) at posttreatment (primary end point), *P* = .03. OLP effects on pain intensity were not significant when testing throughout the entire follow-up period, although there was a marginal effect of OLP at 1-year follow-up (Table 2). Lines reflect sample means and error bars show within-subject SEM. B-F, OLP vs usual care effect sizes on secondary patient-reported outcomes. Effect sizes were computed as group differences in change from baseline to the given time point (Hedges *g*), with negative effects indicating greater improvement for OLP vs usual care. Error bars depict SE for the OLP vs usual care effect size, adjusting for baseline scores.

Open-label placebo led to significant reductions in reported CBP intensity at posttreatment relative to usual care (β = 0.61 points on the 11-point pain scale, *t*[90.09] = 2.29; *P* = .02; with Hedges *g* = 0.45; 95% CI, −0.89 to 0.04) (Figure 2A). Of 44 patients randomized to OLP followed up at post-treatment, 20 (45.4%) reported 30% pain reduction and 11 (24.4%) reported 50% pain reduction. Of 47 patients randomized to usual care followed up at post-treatment, 18 (38.3%) reported 30% pain reduction and 7 (14.9%) had a 50% pain reduction.

Among secondary outcomes at posttreatment, OLP vs usual care led to improvements in pain interference (β = 0.67; *t*[90.58] = 2.65; *P* = .01) and marginal improvements in anxiety (β = 1.38; *t*[91.17] = 1.80; *P* = .08). No significant effects were found at posttreatment for other secondary outcomes (all *P* > .10).

At 1-year follow-up, there were no significant effects of OLP vs usual care on pain intensity, indicating an attenuation of the improvements observed at post-treatment. Benefits of OLP vs usual care were observed at long-term follow-up for depression, anger, anxiety, sleep, global impression of change, and treatment satisfaction questionnaire (all outcomes significant at *P* < .03) (Table 2). Effect sizes at 1-year follow-up were medium sized, ranging from 0.3 to 0.5 (Table 2; eTable 1 in Supplement 2). There were no significant interactions between treatment assignment and time for any outcome (all *P* > .05), suggesting relatively stable effects of treatment throughout the 1-year follow-up period; this was supported by visual inspection of effect size trajectories over time (Figure 2). No adverse effects of treatment were reported by participants at any point. Greater levels of pain catastrophizing at baseline predicted enhanced response to OLP, whereas baseline treatment expectations, trait optimism, anxiety, and depression did not predict OLP response (eMethods, eFigures 2-5, and eResults in Supplement 2).

**Table 2.** Effects of OLP vs Usual Care Through the 1-Year Follow-Up Period

Outcome	Estimate (SE)^a^	β Estimates^a^	*P* value^a^	Effect size at 1 y, Hedges *g* (95% CI)^b^
Pain intensity^c^	−0.41 (0.27)	−1.53	.13	−0.33 (−0.80 to 0.12)^d^
Secondary outcomes				
Pain interference^c^	−0.53 (0.28)	−1.91	.06	−0.30 (−0.74 to 0.09)
Depression^e^	−1.68 (0.54)	−3.13	.002	−0.50 (1.04 to 0.05)^f^
Anger^e^	−1.25 (0.50)	−2.53	.01	−0.38 (−0.85 to 0.05)^f^
Anxiety^e^	−1.77 (0.73)	−2.43	.02	−0.40 (−0.85 to 0.07)^f^
Sleep disruption^e^	−2.11 (0.78)	−2.70	.01	−0.46 (−1.08 to 0.02)^f^
Patient Global Impression of Change^g^	0.69 (0.31)	2.21	.03	0.18 (−0.28 to 0.69)
Treatment Satisfaction Questionniaire^h^	10.73 (4.96)	2.16	.03	0.44 (−0.00 to 0.94)^f^

Abbreviation: OLP, open-label placebo.

^a^ Open-label placebo injection vs usual care led to improvements in multiple patient-reported outcomes in models testing effects across the entire 1-year follow-up period. Data were centered at 6 months, the midpoint of the follow-up time period. To aid interpretation, β estimates are presented in raw units.

^b^ Point estimates and 95% CIs of OLP vs usual care effect sizes at the 1-year follow-up time point. Values for each outcome at each time point are provided in eTable 1 in Supplement 2.

^c^ Brief Pain Inventory–Short Form (scale range, 0 [none] to 10 [worst imaginable]).

^d^ Significant at *P* < .10.

^e^ Patient-Reported Outcomes Measurement Information System for depression (scale range, 0 [none] to 24 [worst]), anger (scale range, 0 [none] to 20 [worst]), anxiety (scale range, 0 [none] to 32 [worst]), and sleep disruption (scale range, 0 [none] to 32 [worst]).

^f^ Significant at *P* < .05.

^g^ Patient Global Impression of Change (scale range, 0 [no improvement] to 7 [largest improvement]).

^h^ Treatment satisfaction (scale range, 0 [no satisfaction] to 100 [highest satisfaction]).

### fMRI Findings

#### Evoked Back Pain Analyses

**Figure 3.**
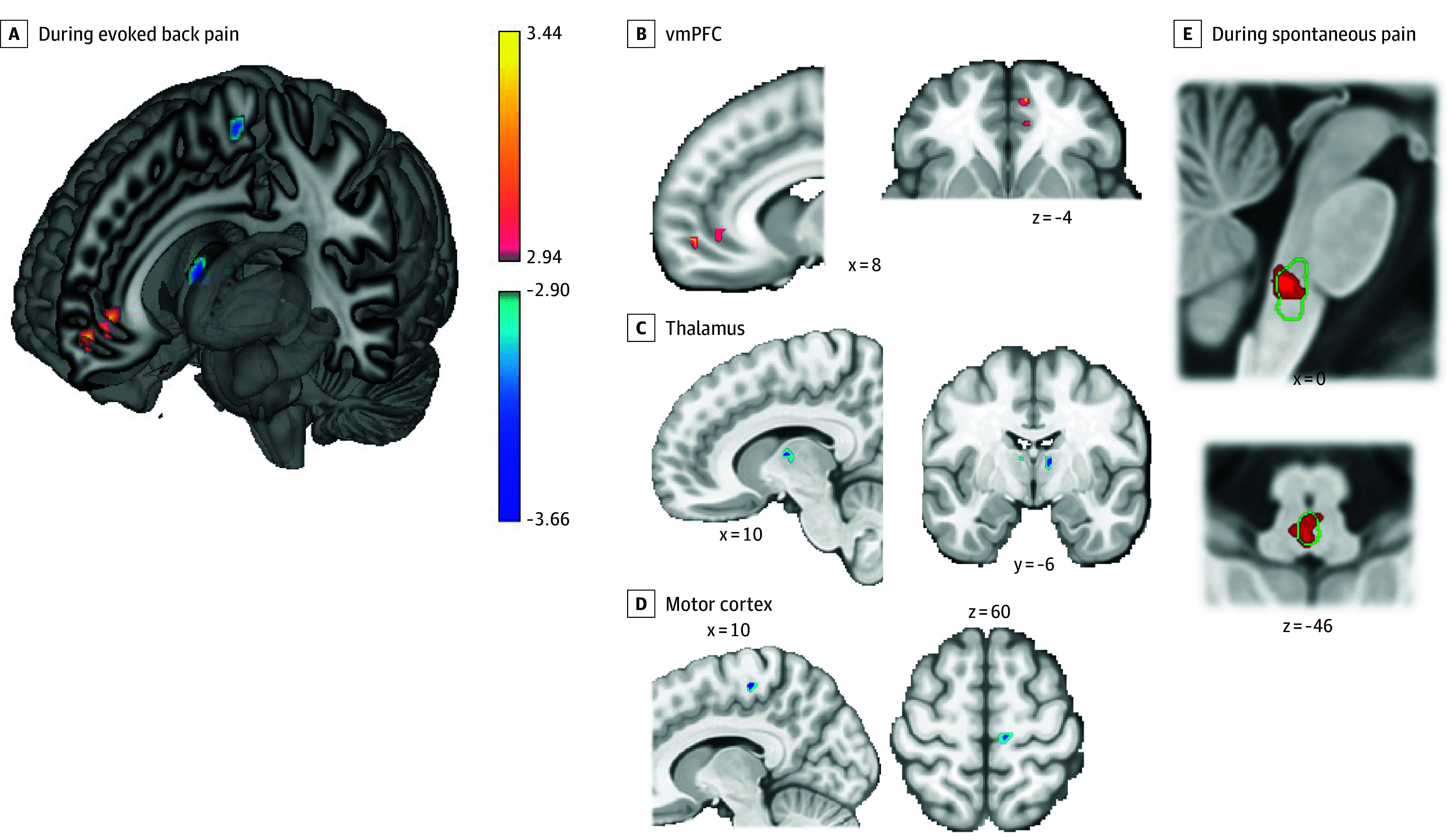
Effects of Open-Label Placebo (OLP) vs Usual Care on Brain Function in Chronic Back Pain A, During evoked back pain, OLP vs usual care led to increased activity in the ventromedial prefrontal cortex (vmPFC) (red/yellow) and decreased activity in primary motor cortex and thalamus (blue), familywise error (FWE) *P* < .05 corrected. Insets show findings for vmPFC (B), thalamus (C), and motor cortex (D). E, During spontaneous pain (resting state), OLP vs usual care led to increased functional connectivity between the more anterior vmPFC region and the rostral ventral medulla (RVM), a brainstem nucleus involved in pain processing and modulation (FWE *P* < .05). Green outlines show RVM location, with vmPFC connectivity increases shown in red. Color bar indicates T statistics; xyz coordinates are in Montreal Neurological Institute space.

Open-label placebo vs usual care led to reduced pain ratings in the back pain evocation task with marginal significance (β = −6.97 on the 0- to 100-point pain scale; *t*[78] = −1.84; *P* = .07). We observed OLP vs usual care increases in evoked back pain–related activity in the vmPFC and rACC and decreases in medial motor cortex (area 4) and thalamus, all FWER-corrected *P* < .05 within ROIs. In addition, the overall combined test showed significant joint effects corrected across all ROIs tested (all FWER-corrected *P* < .05) (Figure 3). No effects were observed in the midcingulate, insula, or dlPFC. The thalamic clusters were labeled as ventral anterior and ventral lateral thalamus, with a predominantly prefrontal connectivity profile in the University of Oxford Thalamic Connectivity Atlas.^47,48^

#### Functional Connectivity During Spontaneous Pain

Of the 2 vmPFC/rACC regions with increased OLP vs usual care activity during evoked pain, the more anterior vmPFC region had significantly increased connectivity during spontaneous pain (resting state) with the RVM (FWER-corrected *P* < .05) (Figure 3), along with marginal connectivity increases with the PAG (*P* < .10 corrected).

## Discussion

Placebo treatments for chronic pain often provide as much or nearly as much pain relief as bona fide pills, injections, and surgeries.^1,2,3,4^ Research reporting the efficacy of nondeceptive OLP has upended the belief that placebos require deception, creating a novel path forward for ethical, feasible placebo treatment.^5,8^ Yet, critical questions remain regarding the efficacy, long-term benefits, and mechanisms of OLP treatments. In particular, to our knowledge, the brain mechanisms of an OLP treatment in a clinical population have not been investigated. Herein, in the context of a randomized clinical trial comparing an OLP injection vs usual care, we found (1) reduced pain intensity at 1 month posttreatment, (2) benefits of OLP on multiple secondary outcomes (but not pain intensity) at 1 year, and (3) altered brain responses to evoked back pain and altered functional connectivity during spontaneous pain, consistent with engagement of descending modulatory pain pathways.

The magnitude of pain reductions we observed at posttreatment is nearly identical to the magnitude of a prior trial of OLP for CBP.^17^ Effects on pain were modest in magnitude (pain reduction of 0.61 of an 11-point scale; Hedges *g* = 0.45) but can be considered clinically significant: many standard CBP treatments (eg, nonsteroidal anti-inflammatory drugs, epidural steroid injections) yield comparable effect sizes but with more adverse events.^2,3^ Another study of OLP for CBP reported larger pain reductions, suggesting that OLP effects may be magnified in certain contexts.^16^

Open-label placebo vs usual care pain intensity reductions were not significant through 1 year follow-up. This is consistent with a study including 3-year follow-up of OLP for CBP^28^ and parallels the effects of epidural steroid injections, whose benefits also typically fade with time. Patients thus often return for repeat steroid injections, although these must be limited due to safety concerns. As there are no safety concerns with repeated OLP injections, future studies could investigate repeated OLP injections as a maintenance treatment aiming to provide sustained pain reductions, with randomized withdrawal studies to estimate the effects of OLP discontinuation.

Sustained benefits of OLP vs usual care through 1-year of follow-up were observed on depression, anxiety, sleep, and anger. These effects were not significant at 1 month posttreatment but emerged later. The delayed emergence of these effects could potentially be explained by mutually reinforcing improvements across these multiple processes (sleep, mood) creating positive feedback loops providing increasing benefits over time, following an initial incubation period.^49^ As a prior trial found limited benefits of OLP vs usual care on depression, stress, and disability at 3 years, these benefits may fade between years 1 and 3 posttreatment.^28^

During evoked back pain, we found OLP vs usual care increases in 2 prefrontal regions, the vmPFC and rACC, as well as decreases in primary motor cortex and thalamus. These results are broadly consistent with investigations of placebo effects on experimental pain in healthy volunteers that have found activations in prefrontal pain-regulatory regions and reductions in somatomotor and nociception-related regions (with substantial variation in specific findings from study to study).^18,19,20,21,22,23,24,25^ During spontaneous pain, we observed increased connectivity between the vmPFC and the RVM, a brainstem nucleus involved in pain modulation.^23,50,51^ Increased vmPFC connectivity to the PAG and RVM has been reported in studies of placebo analgesia in healthy volunteers.^25,45^ It suggests engagement of descending opioidergic projections from the prefrontal cortex to these brainstem nuclei and down to the dorsal horn of the spinal cord, inhibiting afferent nociceptive signals before they reach the brain.^24,50^ Prior experimental work has reported that OLP effects in a laboratory context are partially blocked by naloxone, an opioid antagonist, consistent with the notion that OLP engages opioidergic mechanisms.^52^ As the RVM also includes ascending nociceptive pathways and encodes aversive prediction errors, other interpretations of the increased connectivity are possible as well.^53^ As we observed this increased vmPFC-brainstem coupling during the resting state (spontaneous pain), this raises the possibility that OLP relieves back pain by increasing tonic opioid release in daily life. Overall, these findings suggest that OLP for chronic pain may engage similar brain mechanisms as deceptive placebo for experimental pain, including engagement of prefrontal pain-regulatory regions with projections to brainstem nuclei and reduced activity in nociceptive target regions. To our knowledge, only 2 studies have examined OLP effects on brain function, both examining emotional distress induced by aversive images in healthy volunteers; 1 study reporting increased PAG activity aligned with our findings.^54,55^

Open-label placebo intervention effects were not associated with the inert injection per se, but by the psychosocial context surrounding the injection. The psychological components of the OLP intervention (eg, specific patient education) are likely central to its therapeutic effects.^56,57^

### Limitations

Limitations of the trial include a small sample size, a sample low in racial and ethnic diversity, baseline group differences in exercise levels and pain duration, and more missing data in the usual care arm at 12-month follow-up. As brainstem imaging is methodologically challenging, dedicated fMRI sequences would improve signal strength and localization.^23^ Recruitment materials describing a mind-body intervention may have biased the sample toward people open to accepting a placebo intervention; future research would be needed to test whether openness toward an OLP intervention influences its efficacy.

## Conclusions

In this randomized clinical trial, a placebo injection without deception reduced CBP intensity for 1 month posttreatment and provided benefits lasting for at least 1 year posttreatment. Brain mechanisms of OLP in a clinical population overlapped with those of deceptive placebos in healthy volunteers, including engagement of prefrontal-brainstem pain modulatory pathways.

## Notes

Supplement 1.
Trial Protocol

Supplement 2. **eMethods.** Detailed Methods **eResults.** Detailed Results **eTable 1.** Effects of OLP vs Usual Care on Patient-Reported Outcomes at Each Time point Through 1-Year Follow-Up **eTable 2.** Effects of OLP vs Usual Care on Evoked Back Pain–Related Brain Activity **eFigure 1.** ROIs Tested for OLP vs Usual Care Effects **eFigure 2.** Evoked Back Pain at Pre-treatment **eFigure 3.** Continuous Pain Regressors for Four Randomly Chosen Sample Subjects **eFigure 4.** High vs Low Thumb Pressure Stimulation, FDR q <.05, Serving as a Positive Control **eFigure 5.** Histogram of Quality Control-Functional Connectivity (QC-FC) Correlations for Spontaneous Pain Scans
eReferences

Supplement 3.
Data Sharing Statement
