# Supplementary material for: Open-Label Placebo Injection for Chronic Back Pain With Functional Neuroimaging: A Randomized Clinical Trial
Source: JAMA Netw Open. 2024 Sep 11;7(9):e2432427. doi: 10.1001/jamanetworkopen.2024.32427 (PMC11391328; doi:10.1001/jamanetworkopen.2024.32427)
Supplement: Supplement 2. — eMethods. Detailed Methods eResults. Detailed Results eTable 1. Effects of OLP vs Usual Care on Patient-Reported Outcomes at Each Time point Through 1-Year Follow-Up eTable 2. Effects of OLP vs Usual Care on Evoked Back Pain–Related Brain Activity eFigure 1. ROIs Tested for OLP vs Usual Care Effects eFigure 2. Evoked Back Pain at Pre-treatment eFigure 3. Continuous Pain Regressors for Four Randomly Chosen Sample Subjects eFigure 4. High vs Low Thumb Pressure Stimulation, FDR q <.05, Serving as a Positive Control eFigure 5. Histogram of Quality Control-Functional Connectivity (QC-FC) Correlations for Spontaneous Pain Scans eReferences [file jamanetwopen-e2432427-s002.pdf]

Supplementary Online Content

Ashar YK, Sun M, Knight K, et al. Open-label placebo injection for chronic back pain with functional neuroimaging: a randomized clinical trial. *JAMA Netw Open*. 2024;7(9):e2432427. doi:10.1001/jamanetworkopen.2024.32427

eMethods. Detailed Methods

eResults. Detailed Results

eTable 1. Effects of OLP vs Usual Care on Patient-Reported Outcomes at Each Timepoint Through 1-Year Follow-Up

eTable 2. Effects of OLP vs Usual Care on Evoked Back Pain–Related Brain Activity

eFigure 1. ROIs Tested for OLP vs Usual Care Effects

eFigure 2. Evoked Back Pain at Pre-treatment

eFigure 3. Continuous Pain Regressors for Four Randomly Chosen Sample Subjects

eFigure 4. High vs Low Thumb Pressure Stimulation, FDR q < .05, Serving as a Positive Control

eFigure 5. Histogram of Quality Control-Functional Connectivity (QC-FC) Correlations for Spontaneous Pain Scans

eReferences

This supplementary material has been provided by the authors to give readers additional information about their work.

© 2024 Ashar YK et al. *JAMA Network Open.*

eMethods. Detailed Methods

Exclusion criteria. Exclusion criteria, determined by self-report on the online pre-screen, targeted participants with primary (centralized) CBP. We excluded people with self-reported physician-diagnosed inflammatory disorders, unexplained unintended weight loss of 20 lbs. or more in the past year, and self-reported inability to control bowel or bladder function (a potential indicator of cauda equina syndrome), in addition to the inclusion/exclusion criteria reported in the main text. We also excluded participants unable or unlikely to comply with study procedures: people with self-reported diagnoses of schizophrenia, multiple personality disorder, or dissociative identity disorder; self-reported use of intravenous drugs; difficulty participating for technical/logistical issues (e.g., unable to get to assessment sessions or to complete remote surveys); pain-related compensation or litigation in the past year; and inability to undergo MRI (standard safety screen). People with self-reported history of stroke, brain surgery, or brain tumor were excluded due to difficulties normalizing such brains to standard templates. We also excluded a small number of participants who did not report increased pain during stimulation with a back pain evocation device (described below), because evoked pain was required for our planned fMRI analyses. Numbers excluded are provided in main text Figure 1.

Clinical Measures. We chose average pain intensity over the past week (measured with the Brief Pain Inventory-Short Form) as our primary outcome for several reasons: 1) It is highly interpretable to a broad audience,[1] 2) it is endorsed as a clinical outcome measure by both the IMPAACT committee for low back pain research and the creators of the BPI,[2–4] and 3) it correlated strongly with mean BPI-SF scores in our data (r ~= .90 at each timepoint).

Secondary outcomes measures included: the BPI pain interference,[5] PROMIS short forms for depression (form 8a), anxiety (form 8a), sleep disturbance (form 8a), and anger (form 5a),[6,7] the Patient Global Impression of Change scale (PGIC), and the Treatment Satisfaction Questionnaire.[8]

All measures were collected using the REDCap data collection system. At the pre- and post-treatment assessment sessions, participants completed these measures in a behavioral testing room with no investigators present. Follow up measures were collected remotely using REDCap survey links sent via email or SMS.

Sample size and power analyses. Power analysis determined sample size using a meta-analytic estimate of Cohen's d = .62 for CBP pain intensity for psychological treatment vs. treatment-as-usual.[9] Effects of this size require n = 43 per group to achieve 80% power at α =

© 2024 Ashar YK et al. *JAMA Network Open.*

.05. We aimed to enroll 50 per group, accounting for anticipated attrition, with n = 50 or 51 patients ultimately randomized to each group.

Randomization. Patients were randomized to study and to treatment vs. control using an imbalance-minimization (matching) algorithm,[10] which balanced groups in number and on four covariates: pain intensity, age, gender, and opioid use (yes/no). Half of the control patients came from a simultaneous parallel study with an identical control arm (but testing a different treatment; see registered trial protocol for more details). Randomization and patient notification of group assignment was performed by YA, who had no patient contact during data collection, and group assignment was concealed from research assistants conducting data collection.

MRI acquisition parameters. Structural images were acquired using a single shot T1 MPRAGE sequence with repetition time = 2.4 s, echo time = 2.07 ms, flip angle = 8°, number of slices = 224, slice orientation = sagittal, voxel size = 0.8 mm isotropic, field of view = 256 × 256 mm^2, GRAPPA acceleration factor = 2; echo spacing = 7.6 ms; bandwidth = 240 Hz per pixel.

Functional images were acquired using a multiband gradient-echo EPI sequence with repetition time = 460 ms, multi-band acceleration factor = 8, echo time = 27.2 ms, flip angle = 44°, number of slices = 56, slice orientation = transversal, phase encoding = posterior to anterior, voxel size = 2.7 mm isotropic, gap between slices = 0 mm, field of view = 220 × 220 mm^2, echo spacing = 0.49 ms, bandwidth = 3,048 Hz per pixel.

Evoked back pain. During fMRI, participants completed an evoked back pain task with a series of randomly ordered trials distending the back to one of four intensity levels. The evoked back pain task utilized a novel device providing experimental control over back pain during fMRI. Participants lay on a pneumatically-controlled cylindrical balloon, with increasing inflation causing increasingly painful back distention. The inflatable cylindrical balloon was placed under participants' lower back immediately superior to the iliac crest. Each subject received 20 trials (37 sec duration) at one of four inflation levels and rated pain after each trial on a visual analog scale (VAS; 0 = no pain, 100 = worst pain imaginable), with a total run duration of ~16 minutes (full task design details provided in eMethods p. 3).

fMRI tasks design. Functional scans included an evoked back pain task, a "spontaneous pain" scan, and a thumb pressure-pain task serving as a positive control task for data quality assessment (see fMRI data quality assessment, below).

Evoked back pain task. The evoked back pain task utilized a novel device providing experimental control over back pain during fMRI. Participants lay on a pneumatically-controlled cylindrical balloon, with increasing inflation causing increasingly painful back distention. The inflatable cylindrical balloon was placed under participants' lower back immediately superior to

© 2024 Ashar YK et al. *JAMA Network Open.*

the iliac crest. Distance from the balloon to the lateral malleolus was measured at pre-treatment and the balloon was placed was in the same location at post-treatment. Each subject received 20 trials (37 sec duration) at one of four inflation levels, and patients rated post-trial pain on a visual analog scale (VAS; 0 = no pain, 100 = worst pain imaginable). The balloon was never fully deflated during this task to limit larger head motions. The order of inflation levels for each subject was randomly permuted but constrained to optimize design efficiency by avoiding correlation with low frequency signals: Trials of inflation level 1 or 2 were always followed by inflation level 3 or 4 and vice versa, and consecutive trials always had different inflation levels. We adopted an extensive set of strategies for mitigating and controlling for head motion, described below. The evoked back pain scan was missing for one subject at post-treatment due to technical issues. Pain ratings from the evoked back pain task are shown in eFigure 2.

Spontaneous pain (resting state) scan. Participants completed a scan measuring spontaneously occurring pain (no stimulation). Participants fixated on a foveal crosshair and provided a VAS rating (7 sec) of current back pain intensity each minute. We regressed out the rating task and analyzed residual resting-state connectivity, as described below.

MRI preprocessing pipeline. Standard fMRI preprocessing procedures were used, implemented in *fMRIprep* 1.2.4[11] which is based on Nipype 1.1.6.[12] Anatomical T1-weighted (T1w) images from both scanning sessions were corrected for intensity non-uniformity (INU) sing N4BiasFieldCorrection[13] (ANTs 2.2.0). A T1w-reference map was computed after registration of the two T1w images (after INU-correction) using mri_robust_template.[14] The T1w-reference was then skull-stripped using antsBrainExtraction.sh (ANTs 2.2.0), using OASIS as target template. Spatial normalization to the ICBM 152 Nonlinear Asymmetrical template version 2009c[15] was performed through nonlinear registration with antsRegistration, using brain-extracted versions of both the T1w volume and the template.

For the functional run, first a reference volume and its skull-stripped version were generated using a custom methodology of fMRIPrep. A deformation field to correct for susceptibility distortions was estimated based on two echo-planar imaging (EPI) references with opposing phase-encoding directions, using 3dQwarp[16] (AFNI 20160207). Based on the estimated susceptibility distortion, an unwarped BOLD reference was calculated for a more accurate co-registration with the anatomical reference. The BOLD reference was then co-registered to the T1w reference using flirt[17] (FSL 5.0.9) with the boundary-based registration cost-function.[18] Co-registration was configured with nine degrees of freedom to account for distortions remaining in the BOLD reference. Head-motion parameters were estimated with respect to the BOLD reference before any spatiotemporal filtering

© 2024 Ashar YK et al. *JAMA Network Open.*

using mcflirt (FSL 5.0.9). The BOLD time-series were resampled onto their original, native space by applying a single, composite transform to correct for head-motion and susceptibility distortions. The BOLD time-series were resampled to MNI152NLin2009cAsym standard space, generating a preprocessed BOLD run in MNI152NLin2009cAsym space using antsApplyTransforms, configured with Lanczos interpolation to minimize the smoothing effects of other kernels.[19]

Denoising pipeline. For both the evoked and spontaneous pain scans, nuisance covariates included 24 head motion parameters and "spike" regressors identifying volumes with framewise displacement (FD) >= .25 mm (often considered a strict threshold[20]). Spike regression was optimized for fast-TR data by a) applying a [.1 Hz – .5 Hz] band-stop filter to head motion parameters prior to computing FD, and b) computing FD with respect to the volume collected 2.4 sec previously (5 volume difference).[21,22] We additionally included spike regressors for the four volumes following an identified spike, since effects of head motion can influence subsequent volumes as well. These nuisance covariates were included in 1st level models for evoked pain analyses and were regressed out of the spontaneous pain scans prior to connectivity analyses.

Denoising for the evoked back pain task included two additional procedures to limit the influence of head motion and remove signal less likely to be of neuronal origin. We applied anatomical CompCor, which generates nuisance covariates derived from signal fluctuations in white matter and cerebrospinal fluid,[23] and we included nuisance covariates with signal timeseries extracted from an anterior and a posterior out-of-brain area, to further capture and remove artifactual signal fluctuations in the data.

Denoising for spontaneous pain connectivity analyses additionally included global signal regression and band-pass filtering [.1 – .01 Hz], to focus on signal fluctuations most likely to be of neuronal origin.[24] We also included a nuisance regressor modelling the pain rating task (boxcar regressor) in order to more closely resemble traditional resting state analyses, though prior work has found that intrinsic connectivity networks are strongly preserved during the task performance.[25]

Model of continuous evoked pain. To limit the confounding of pain-related and rating-related neural signals, we collected only brief post-trial ratings (VAS, 7 sec). Since participants were in pain throughout the task, we developed an exponential decay model of continuous pain based on the post-trial ratings.

This model was validated on a separate data set collected on a subset of study participants (*n* = 58) who completed an evoked back pain task in a behavioral testing room

during their eligibility session visit. The validation task was identical to the task administered during fMRI, except participants provided continuous pain ratings using a trackball rather than brief post-trial ratings as during fMRI.

We fit an exponential decay model to estimate continuous pain between post-trial ratings, modelling a more rapid change in pain at the beginning of each trial followed by an asymptotic approach to the next sample point. The model fit the formula $f(x) = (b-a) * (1 – e^\tau*x) + a$, with a = pain rating at trial start, b = pain rating at trial end, x = trial timepoints between samples, and τ = a time constant governing the exponential decay process. τ was fit for each trial using the MATLAB curve fitting toolbox, and the average τ value across all subjects' trials was used to assess model performance (R^2 with bootstrapped confidence intervals, 10,000 bootstrap samples, MATLAB *bootci* function).

In this validation task, reported and model-predicted continuous pain were strongly related, mean R^2 = .85, 95% CI = [.82 .87]. Exponential decay model fits for four sample subjects are shown in eFigure 3.

Scans were excluded from analyses if pain ratings were missing on >= 25% of trials (n = 12), almost no pain was reported (pain <= 5/100 on 90% of trials, n = 3), or there was insufficient variability in pain (range <= 10/100, n = 3), as pain could not be reliably modelled in these scans.

Relationships with evoked back pain were identified by constructing a continuous within-person estimate of evoked pain intensity based on post-trial pain ratings (continuous evoked pain model, described below). Continuous pain values were entered as a regressor in each subject's 1st-level model (Z-scored so that voxel parameter estimates would capture pain magnitude) along with nuisance covariates, to estimate an evoked back pain parametric map for each participant at pre-treatment.

fMRI data quality assessment. For the evoked back pain task, we assessed the influence of head motion at both the within-subject and between-subject level. Within-subject, we computed the variance inflation factor (VIF) for the pain regressor relative to the 24 head motion parameters, providing an estimate of task-correlated head motion. At the between-subject level, we included a head motion summary statistic (number of volumes identified as motion outliers) as a covariate in the mixed effects model to and we tested for PRT vs. TAU differences in head motion.

For resting connectivity analyses with the spontaneous pain scans, we assessed data quality with "quality control-functional connectivity" ("QC-FC") correlations, using a whole-brain parcellation that included 489 parcels.[26,27] We computed the distribution of correlations between

© 2024 Ashar YK et al. *JAMA Network Open.*

connectivity estimates and head motion across edges, an established measure of head motion associations with connectivity estimates.[20,24,28] We also tested for PRT vs. TAU group differences in head motion and repeated the 2nd level models with a head motion covariate. The median edge connectivity value was computed, and subjects' >= 3 standard deviations above the mean were excluded.

We also used a positive control task for data quality assessment. We administered 20 thumb pressure stimulations at a high and low pressure (4 and 7 kg/cm^2), estimated the [high – low] contrast for each subject, and applied an FDR $q < .05$ threshold to the group average contrast map using standard general linear model analyses in SPM12. We expected significant associations with the brain responses reliably reported in acute pain tasks (e.g., midcingulate, thalamus, insula, somatosensory cortex, cerebellum).

Region of interest (ROI) definitions. ROI boundaries were defined anatomically (thalamus, PAG, RVM), from NeuroSynth (vmPFC, reverse inference map), or as spheres (16 mm radius) around peak coordinates from a previous individual patient meta-analysis of placebo analgesia.[29] ROIs were chosen to include regions relevant for evoked back pain and reliably shown to exhibit placebo-induced *increases* (vmPFC, dlPFC) or placebo-induced *decreases* (thalamus, insula, cingulate, somatomotor cortex) in activity in prior reviews and meta-analyses.[29–32]

Permutation testing approach. We applied a two-stage combination permutation test, as described in Winkler et al.[35] The first stage involves small volume correction within each of a series of ROIs. The second stage is a joint test of signal across ROIs to correct for the number of ROIs tested. We used a threshold of $p < 0.05$ Familywise Error Rate (FWER) corrected for both stages.

In Stage 1, within each ROI, we computed the *exceedance mass* for the observed data and compared it to a null distribution created by 5,000 permutations of the group labels (OLP vs. Usual Care). Exceedance mass was defined as the sum of the voxelwise T statistics exceeding a prespecified cluster-defining threshold. Because the choice of cluster-defining threshold is arbitrary, we confirmed that our results held across three different cluster-defining thresholds (p = .05, .01, and .005). When the exceedance mass for the observed (unpermuted) data exceeded the 95th percentile of the null distribution (for regions hypothesized to exhibit increases) or fell below the 5th percentile of the null distribution (for regions hypothesized to exhibit decreases), findings were considered significant. Directional hypotheses were provided by prior placebo analgesia meta-analyses finding increased prefrontal activity and decreased activity in nociceptive and somatosensory regions and were built in to our theoretical framework

© 2024 Ashar YK et al. *JAMA Network Open.*

for selection of ROIs.[29–32] Use of the exceedance mass as a maximal statistic in permutation testing provides greater sensitivity than single-voxel statistics (e.g., voxelwise max T) and cluster size (number of suprathreshold voxels), as exceedance mass integrates both cluster height and extent.[33,34]

In Stage 2, to correct across the six ROIs tested, we constructed a combined statistic based on Fisher's method for combining *p* values, and we compared this statistic to its null distribution based on the permuted data. For each permutation, we computed a null-hypothesis *p* value for each ROI as the rank order of that permutation's exceedance mass statistic within the null distribution, and we combined those *p* values across the six ROIs using Fisher's formula (-2 * sum(log(p)). This provided a null distribution of the combined test statistic across ROIs.[35] The observed Fisher-combined test statistic was compared to the 95% percentile of the null distribution of combined test statistics, providing a non-parametric test of whether there was more signal than expected by chance across ROIs.[35]

Our models comparing OLP vs. UC used different covariates for each voxel (the pre-treatment evoked pain response in that voxel), as this is recommended by statisticians over pre-to-post-intervention change scores for detecting intervention effects.[36,37] Due to having different covariates for each voxel, the FSL PALM toolbox for non-parametric combination tests could not be applied for our use case.

Exploratory whole-brain results. To conduct a sensitivity analysis investigating whether our ROI-based approach missed signal elsewhere in the brain, we conducted a whole-brain analyses thresholded at $p < .005$ uncorrected, $k = 10$ mm, with results presented below.

Anatomical labeling of regions and assessment of functional connectivity profiles. Regions were labeled using the CANLab Combined 2024 Atlas (https://sites.google.com/dartmouth.edu/canlab-brainpatterns/brain-atlases-and-parcellations/2018-combined-atlas) which integrates multiple published cortical and subcortical atlases and by reference to the Julich histological atlas.[38] First- and second-level models and functional connectivity profiles (see below) were estimated using SPM12 and the CanlabCore toolbox (https://github.com/canlab/CanlabCore).

To help interpret thalamic results, we visually examined the mean functional connectivity profiles of resulting clusters by estimating their functional connectivity in our data at pre-randomization and quantified their probabilistic connectivity profile in the Oxford Thalamic Connectivity Atlas.[39]

Computation of effect sizes for clinical outcomes. We computed effect sizes (Hedges' *g*) of OLP vs. Usual Care group differences at each timepoint in the 1-year follow-up

© 2024 Ashar YK et al. *JAMA Network Open.*

period (change from baseline to the given timepoint, confidence intervals based on 1000 bootstrapped samples). As PGIC and Treatment Satisfaction scores were not provided at baseline, effect sizes for these measures were computed as OLP vs. Usual Care group differences at the given timepoint.

Baseline predictors of response to OLP. For archival purposes, we tested whether baseline measures of psychological functioning predicted response to OLP. We examined the following measures: pain catastrophizing (Pain Catastrophizing Scale), a one-item measure of expectations adapted from the CEQ (“At this point, how successful do you think this treatment will be in reducing your back pain?”; visual analog scale ranging from “Not at all successful” to “Very successful”), depression and anxiety (PROMIS short forms), optimism (Revised Life Orientation Test, LOT-R), and duration of chronic back pain (in years). These measures were collected at pre-randomization. For the expectation item, we asked participants to answer as if they had been randomized to treatment.

We first tested each measure as a moderator, predicting differential response to OLP vs. Usual Care. We estimated a GLM predicting post-treatment pain intensity from pre-treatment pain intensity, the measure of interest, group, and the group *x* measure interaction. We then tested whether each measure of interest predicted response among OLP participants only by estimating a GLM predicting post-treatment pain intensity from pre-treatment pain intensity and the measure of interest in the OLP group.

© 2024 Ashar YK et al. *JAMA Network Open.*

eResults. Detailed Results

fMRI data quality assessment. The positive control task produced the expected activations in pain-responsive regions (eFigure 4). Spontaneous pain scan correlations between head motion and functional connectivity estimates ("QC-FC correlations") were low, $r = .02$ (SD = .19) across edges (eFigure 5).[20,24,28] There were no group differences in head motion at pre- or post-treatment, both $p > .55$. One subject was excluded from spontaneous pain connectivity analyses due to poor data quality (median edge correlation more than 3 standard deviations above the mean).

In the evoked back pain task, subjects had M = 189.11 (218.70) volumes flagged as spikes (~12% of volumes). This relatively strict approach to identifying volumes potentially corrupted by head motion still provided M = 11.03 (1.67) min of data for analyses. There were no group differences in head motion at pre- or post-treatment, both $p > .2$. Within-subject assessment of task-correlated head motion found mean VIF = 2.9 (SD = 1.5). Only one subject had VIF >= 10, a commonly used threshold for high collinearity. There were no group differences in VIFs at either timepoint, both $p > .13$. Overall, this suggests a limited influence of head motion on evoked back pain estimates at both the within- and between-subject level.

Baseline predictors of response to OLP. Greater levels of pain catastrophizing at baseline predicted enhanced response to OLP. This was found in moderator analyses comparing OLP to Usual Care, group *x* catastrophizing $\beta = -0.04$, $t(81) = -2.43$, $p = .017$, and was marginally significant in analyses of OLP participants alone, catastrophizing $\beta = -0.06$, $t(81) = -1.93$, $p = .06$. Depression, anxiety, optimism, expectations, and duration of back pain were not significantly associated with OLP response in moderator analyses or in analyses of the OLP group alone.

Effects of OLP on BPI Severity scale. At the request of an anonymous reviewer, we conducted analyses of Brief Pain Inventory-Short Form Severity scale as an outcome. The BPI Severity scale includes 4 items, measuring last-week average, best, and worst pain, plus current pain. As described above (section Clinical Measures, p. 2) and in our preregistered analytic plan, we chose last-week average pain intensity (the first of these four items) as the primary outcome in this trial. Effects of OLP vs. Usual care on BPI Severity were in the expected direction but not significant, $\beta = 0.33$, $t(91.5) = 1.38$, $p = 0.17$. This indicates that OLP may have attenuated effects on best, worst, and/or current pain relative to last-week average pain. Further research may be needed to better understand the differential effects of treatments on average, best, worst, and current pain.

© 2024 Ashar YK et al. *JAMA Network Open.*

Exploratory whole-brain results. In addition to the results identified in ROI analyses, we observed OLP vs. Usual Care decreases in evoked back pain response in several areas: the right operculum, parahippocampal cortex, premotor cortex, and temporoparietal cortex (eTable 2).

© 2024 Ashar YK et al. *JAMA Network Open.*

.

eTable 1

Effects of OLP vs. Usual Care on patient-reported outcomes at each timepoint through 1-year follow-up.

Measure	OLP mean (SD)	Usual care mean (SD)	Hedges g
**Pain Intensity, 0 - 10**			
Baseline	4.20 (1.27)	3.99 (1.23)	NaN
Post-tx	2.93 (1.61)	3.14 (1.50)	-0.45
1 month	2.95 (2.00)	3.15 (1.63)	-0.41
2 months	3.18 (1.86)	3.11 (1.85)	-0.17
3 months	3.36 (1.96)	3.31 (1.97)	-0.34
6 months	2.83 (2.04)	3.00 (1.97)	-0.28
12 months	2.83 (1.78)	3.09 (1.75)	-0.33
**Pain Interference, 0 - 10**			
Baseline	3.48 (1.64)	3.52 (1.54)	NaN
Post-tx	2.21 (1.88)	2.58 (1.63)	-0.39
1 month	2.15 (1.87)	2.58 (1.68)	-0.56
2 months	2.06 (1.73)	2.67 (1.88)	-0.60
3 months	2.88 (2.41)	2.86 (2.18)	-0.29
6 months	2.28 (2.25)	2.73 (1.84)	-0.40
12 months	2.28 (1.96)	2.66 (2.17)	-0.30
**PROMIS Depression**			
Baseline	13.34 (4.68)	12.85 (4.72)	NaN
Post-tx	11.86 (4.10)	11.68 (4.30)	-0.23
1 month	10.64 (3.61)	11.71 (4.69)	-0.82
2 months	11.24 (5.07)	11.94 (5.26)	-0.51
3 months	12.69 (6.17)	12.49 (4.57)	-0.34
6 months	11.09 (4.08)	12.00 (4.72)	-0.52
12 months	11.98 (5.94)	13.00 (4.51)	-0.50
**PROMIS Anger**			
Baseline	10.93 (3.23)	11.24 (3.12)	NaN
Post-tx	10.05 (3.81)	10.48 (3.69)	-0.12
1 month	8.95 (3.29)	10.63 (3.23)	-0.72
2 months	9.45 (3.30)	10.17 (3.94)	-0.18
3 months	10.06 (4.83)	10.69 (3.50)	-0.29
6 months	9.44 (2.96)	10.71 (3.42)	-0.49
12 months	9.66 (3.60)	11.15 (3.28)	-0.38
**PROMIS Anxiety**			
Baseline	15.79 (5.79)	15.19 (6.36)	NaN

© 2024 Ashar YK et al. *JAMA Network Open.*

Post-tx	14.10 (5.82)	13.98 (6.84)	-0.21
1 month	12.38 (4.87)	14.05 (6.82)	-0.68
2 months	13.45 (6.87)	13.86 (6.83)	-0.28
3 months	14.83 (7.49)	14.29 (6.54)	-0.23
6 months	13.15 (5.22)	14.97 (6.91)	-0.67
12 months	14.15 (7.59)	15.15 (6.99)	-0.40
**PROMIS Sleep**			
Baseline	22.54 (6.47)	22.90 (6.20)	NaN
Post-tx	20.38 (6.19)	21.00 (6.01)	-0.13
1 month	20.88 (6.15)	21.61 (6.56)	-0.41
2 months	19.52 (6.70)	22.06 (7.20)	-0.48
3 months	20.22 (7.10)	22.03 (6.21)	-0.58
6 months	19.35 (6.26)	21.71 (5.93)	-0.58
12 months	19.85 (5.83)	21.35 (6.71)	-0.46
**Patient Global Impression of Change**			
Post-tx	3.62 (1.62)	2.14 (1.47)	0.95
1 month	3.38 (1.67)	2.11 (1.31)	0.83
2 months	3.42 (1.80)	2.64 (1.71)	0.44
3 months	3.25 (1.76)	2.20 (1.37)	0.66
6 months	3.55 (1.77)	2.46 (1.72)	0.62
12 months	3.54 (1.91)	3.18 (1.99)	0.18
**Treatment Satisfaction Questionnaire**			
Post-tx	58.21 (23.41)	36.35 (23.57)	0.92
1 month	53.68 (27.28)	43.74 (18.13)	0.42
2 months	49.61 (29.37)	44.10 (21.28)	0.22
3 months	50.79 (29.76)	39.97 (20.46)	0.42
6 months	54.88 (28.54)	38.64 (24.43)	0.61
12 months	50.89 (30.36)	38.44 (24.41)	0.44

Note. Mean values for patient-reported outcomes for open-label placebo (OLP) and usual care at each timepoint. Effect sizes (Hedge's *g*) indicate effects of OLP vs. usual care in change scores from baseline to the given timepoint. For the patient global impression of change and treatment satisfaction questionnaires, there are no baseline values and effect sizes represent comparisons at the given timepoint.

© 2024 Ashar YK et al. *JAMA Network Open.*

eTable 2

Effects of OLP vs. Usual Care on evoked back pain-related brain activity

Region	X	Y	Z	Brodmann area	Max Z
**OLP vs. Usual Care increases**					
Ventromedial PFC*	6	53	-8	10	3.36
Pregenual cingulate*	9	42	-2	24	3.35
**OLP vs. Usual Care reductions**					
Parahippocampal cortex	-21	-46	-8	ph1	-3.20
Premotor motor	6	-1	52	6ma	-3.49
Primary motor*	11	-25	57	4a	-4.56
Temporoparietal	-45	-46	6	TPJ	-3.57
Parietal operculum	-53	2	19	44	-3.39
Ventral anterior thalamus*	11	-6	8	-	-3.93
Ventral lateral thalamus*	-7	-9	11	-	-3.17

Note. Effects of open-label placebo (OLP) vs. Usual Care on brain responses to evoked back pain. Regions were labeled using a fusion registration projection of the Julich histological atlas[38] to the volumetric space of the normalization template. * indicates results significant at FWE $p < .05$ and depicted in Figure 3; regions not indicated with an asterisk survive an exploratory $p < .005$ uncorrected threshold and are included here for archival purposes.

© 2024 Ashar YK et al. *JAMA Network Open.*

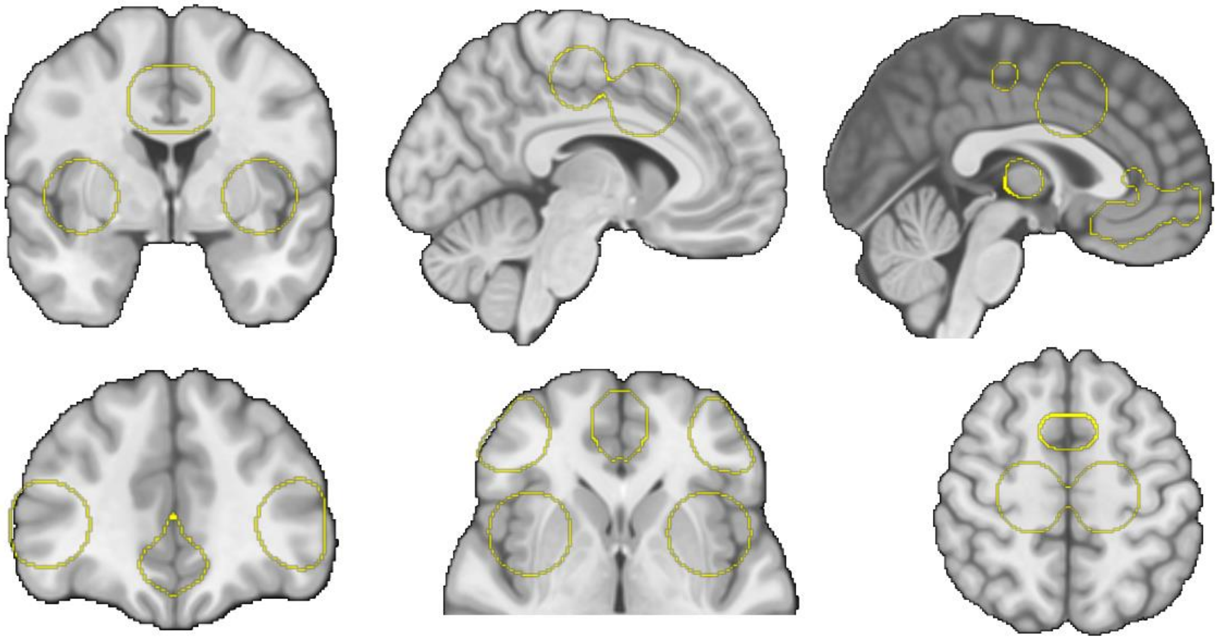

eFigure 1. ROIs tested for OLP vs. Usual Care effects. We searched for OLP vs. Usual Care differences within each region (small volume corrected) and tested the joint significance of effects across regions. See text for details of ROI selection and definition.

© 2024 Ashar YK et al. *JAMA Network Open.*

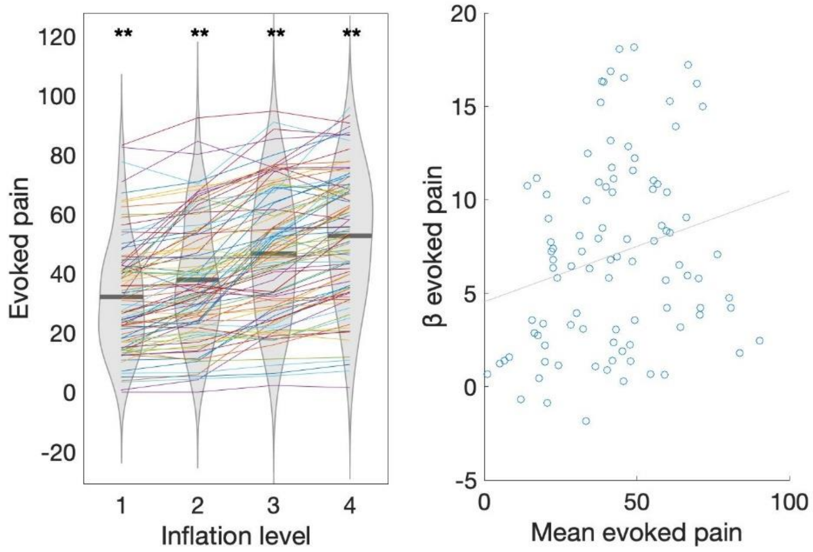

eFigure 2. Evoked back pain at pre-treatment. The left panel shows mean evoked pain at each inflation level for each patient (colored lines). The right panel shows mean evoked pain by increase in pain per increase in inflation level (β evoked pain, estimated using linear regression) for each patient.

© 2024 Ashar YK et al. *JAMA Network Open.*

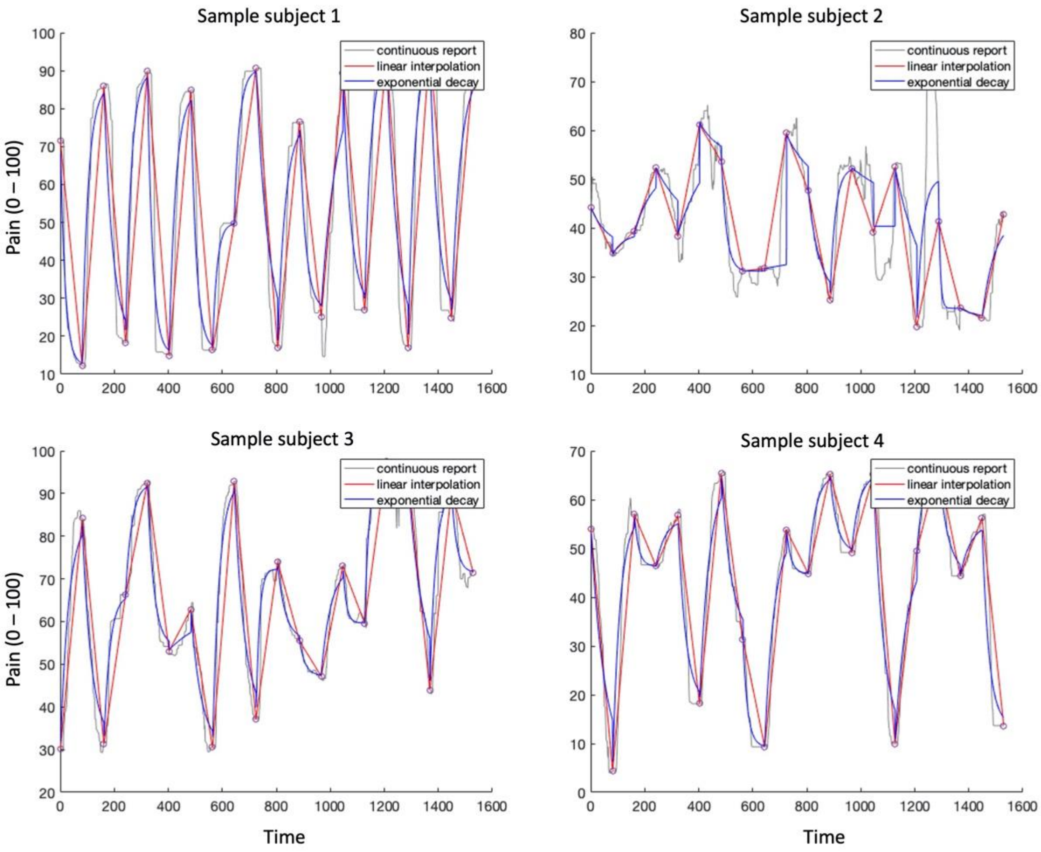

eFigure 3. Continuous pain regressors for four randomly chosen sample subjects. Grey line shows observed continuous report in the validation data, with gray circles indicating the samples taken at post-trial intervals. Predicted continuous pain between samples is shown for the linear interpolation (red) and exponential decay model (blue).

© 2024 Ashar YK et al. *JAMA Network Open.*

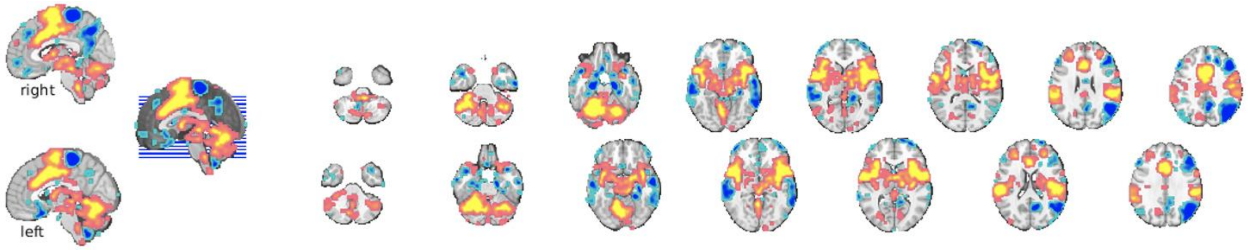

eFigure 4. High vs. low thumb pressure stimulation, FDR $q < .05$, serving as a positive control. Effects are observed in the expected pain-responsive regions.

© 2024 Ashar YK et al. *JAMA Network Open.*

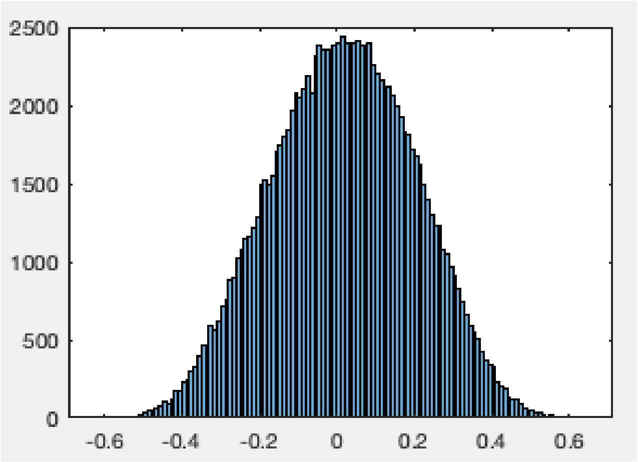

eFigure 5. Histogram of quality control-functional connectivity (QC-FC) correlations for spontaneous pain scans.

© 2024 Ashar YK et al. *JAMA Network Open.*

eReferences

1. Pek J, Flora DB. Reporting effect sizes in original psychological research: A discussion and tutorial. *Psychol Methods*. 2018;23(2):208-225. doi:10.1037/met0000126
2. Dworkin RH, Turk DC, Farrar JT, et al. Core outcome measures for chronic pain clinical trials: IMMPACT recommendations. *Pain*. 2005;113(1-2):9-19. doi:10.1016/j.pain.2004.09.012
3. Ramasamy A, Martin ML, Blum SI, et al. Assessment of Patient-Reported Outcome Instruments to Assess Chronic Low Back Pain. *Pain Medicine*. Published online 2017:1-13. doi:10.1093/pm/pnw357
4. Cleeland C, Ryan K. *The Brief Pain Inventory*. Pain Research Group; 1991.
5. Fairbank JC, Couper J, Davies JB, O'Brien JP. The Oswestry low back pain disability questionnaire. *Physiotherapy*. 1980;66(8):271-273. doi:PMID: 6450426
6. Stone AA, Broderick JE, Junghaenel DU, Schneider S, Schwartz JE. PROMIS fatigue, pain intensity, pain interference, pain behavior, physical function, depression, anxiety, and anger scales demonstrate ecological validity. *J Clin Epidemiol*. Published online November 25, 2015. doi:10.1016/j.jclinepi.2015.08.029
7. Licciardone J, Worzer WE, Hartzell MM, Kishino N, Gatchel RJ. An Overview of the Patient-Reported Outcomes Measurement Information System (PROMIS) for Assessing Chronic Low Back Pain Patients. *J Appl Biobehav Res*. Published online 2017:1-22. doi:10.1111/jabr.12057
8. Atkinson MJ, Sinha A, Hass SL, et al. Validation of a general measure of treatment satisfaction, the Treatment Satisfaction Questionnaire for Medication (TSQM), using a national panel study of chronic disease. *Health Qual Life Outcomes*. 2004;2(1):12. doi:10.1186/1477-7525-2-12
9. Hoffman BM, Papas RK, Chatkoff DK, Kerns RD. Meta-analysis of psychological interventions for chronic low back pain. *Health Psychol*. 2007;26(1):1-9. doi:10.1037/0278-6133.26.1.1
10. Xiao L, Yank V, Ma J. Algorithm for balancing both continuous and categorical covariates in randomized controlled trials. *Comput Methods Programs Biomed*. 2012;108(3):1185-1190. doi:10.1016/j.cmpb.2012.06.001
11. Esteban O, Markiewicz CJ, Blair RW, et al. fMRIPrep: a robust preprocessing pipeline for functional MRI. *Nat Methods*. 2019;16(1):111-116. doi:10.1038/s41592-018-0235-4
12. Gorgolewski K, Burns CD, Madison C, et al. Nipype: A Flexible, Lightweight and Extensible Neuroimaging Data Processing Framework in Python. *Front Neuroinform*. 2011;5:13. doi:10.3389/fninf.2011.00013
13. Tustison NJ, Avants BB, Cook PA, et al. N4ITK: Improved N3 Bias Correction. *IEEE Trans Med Imaging*. 2010;29(6):1310-1320. doi:10.1109/TMI.2010.2046908
14. Reuter M, Rosas HD, Fischl B. Highly accurate inverse consistent registration: A robust approach. *Neuroimage*. 2010;53(4):1181-1196. doi:10.1016/J.NEUROIMAGE.2010.07.020
15. Fonov V, Evans A, McKinstry R, Almli C, Collins D. Unbiased nonlinear average age-appropriate brain templates from birth to adulthood. *Neuroimage*. 2009;47:S102. doi:10.1016/S1053-8119(09)70884-5

© 2024 Ashar YK et al. *JAMA Network Open.*

16. Cox RW, Hyde JS. Software tools for analysis and visualization of fMRI data. *NMR Biomed*. 1997;10(4-5):171-178. doi:10.1002/(SICI)1099-1492(199706/08)10:4/5<171::AID-NBM453>3.0.CO;2-L
17. Jenkinson M, Smith S. A global optimisation method for robust affine registration of brain images. *Med Image Anal*. 2001;5(2):143-156. doi:10.1016/S1361-8415(01)00036-6
18. Greve DN, Fischl B. Accurate and robust brain image alignment using boundary-based registration. *Neuroimage*. 2009;48(1):63-72. doi:10.1016/J.NEUROIMAGE.2009.06.060
19. Lanczos C. Evaluation of Noisy Data. *Journal of the Society for Industrial and Applied Mathematics Series B Numerical Analysis*. 1964;1(1):76-85. doi:10.1137/0701007
20. Power JD, Barnes KA, Snyder AZ, Schlaggar BL, Petersen SE. Spurious but systematic correlations in functional connectivity MRI networks arise from subject motion. *Neuroimage*. 2012;59(3):2142-2154. doi:10.1016/j.neuroimage.2011.10.018
21. Power JD, Lynch CJ, Silver BM, Dubin MJ, Martin A, Jones RM. Distinctions among real and apparent respiratory motions in human fMRI data. *Neuroimage*. 2019;201(July):116041. doi:10.1016/j.neuroimage.2019.116041
22. Snellenberg V. Motion denoising of multiband resting state functional connectivity MRI data: An improved volume censoring method. 2019;c.
23. Behzadi Y, Restom K, Liau J, Liu TT. A component based noise correction method (CompCor) for BOLD and perfusion based fMRI. *Neuroimage*. 2007;37(1):90-101. doi:10.1016/j.neuroimage.2007.04.042
24. Ciric R, Rosen AFG, Erus G, et al. Mitigating head motion artifact in functional connectivity MRI. *Nat Protoc*. 2018;13(12):2801-2826. doi:10.1038/s41596-018-0065-y
25. Cole MW, Bassett DS, Power JD, Braver TS, Petersen SE. Intrinsic and task-evoked network architectures of the human brain. *Neuron*. 2014;83(1):238-251. doi:10.1016/j.neuron.2014.05.014
26. Glasser MF, Coalson TS, Robinson EC, et al. A multi-modal parcellation of human cerebral cortex. *Nature*. Published online 2016:1-11. doi:10.1038/nature18933
27. Pauli WM, Nili AN, Tyszka JM. A high-resolution probabilistic in vivo atlas of human subcortical brain nuclei. *Sci Data*. 2018;5:180063.
28. Parkes L, Fulcher B, Yücel M, Fornito A. NeuroImage An evaluation of the ef fi cacy , reliability , and sensitivity of motion correction strategies for resting-state functional MRI. *Neuroimage*. 2018;171(December 2017):415-436. doi:10.1016/j.neuroimage.2017.12.073
29. Zunhammer M, Spisák T, Wager TD, et al. Meta-analysis of neural systems underlying placebo analgesia from individual participant fMRI data. *Nat Commun*. 2021;12(1):1-11. doi:10.1038/s41467-021-21179-3
30. Ashar YK, Chang LJ, Wager TD. Brain Mechanisms of the Placebo Effect: An Affective Appraisal Account. *Annu Rev Clin Psychol*. 2017;13(1):73-98. doi:10.1146/annurev-clinpsy-021815-093015
31. Wager TD, Atlas LY. The neuroscience of placebo effects: Connecting context, learning and health. *Nat Rev Neurosci*. 2015;16(7):403-418. doi:10.1038/nrn3976
32. Amanzio M, Benedetti F, Porro CA, Palermo S, Cauda F. Activation likelihood estimation meta-analysis of brain correlates of placebo analgesia in human experimental pain. *Hum Brain Mapp*. 2013;34(3):738-752. doi:10.1002/hbm.21471

© 2024 Ashar YK et al. *JAMA Network Open.*

33. Nichols TE, Holmes AP. Nonparametric permutation tests for functional neuroimaging: a primer with examples. *Hum Brain Mapp*. 2002;15(1):1-25. doi:10.1002/hbm.1058
34. Bullmore ET, Suckling J, Overmeyer S, Rabe-Hesketh S, Taylor E, Brammer MJ. Global, voxel, and cluster tests, by theory and permutation, for a difference between two groups of structural mr images of the brain. *IEEE Trans Med Imaging*. 1999;18(1):32-42. doi:10.1109/42.750253
35. Winkler AM, Webster MA, Brooks JC, Tracey I, Smith SM, Nichols TE. Non-parametric combination and related permutation tests for neuroimaging. *Hum Brain Mapp*. 2016;37(4):1486-1511. doi:10.1002/hbm.23115
36. Judd CM, Mcclelland G, Ryan C. *Data Analysis: A Model Comparison Approach*. Routledge; 2009.
37. O Connell NS, Dai L, Jiang Y, et al. Methods for Analysis of Pre-Post Data in Clinical Research: A Comparison of Five Common Methods. *J Biom Biostat*. 2017;08(01):1-8. doi:10.4172/2155-6180.1000334
38. Amunts K, Mohlberg H, Bludau S, Zilles K. Julich-Brain: A 3D probabilistic atlas of the human brain's cytoarchitecture. *Science (1979)*. 2020;369(6506):988-992. doi:10.1126/science.abb4588
39. Behrens TEJ, Johansen-Berg H, Woolrich MW, et al. Non-invasive mapping of connections between human thalamus and cortex using diffusion imaging. *Nat Neurosci*. 2003;6(7):750-757. doi:10.1038/nn1075

© 2024 Ashar YK et al. *JAMA Network Open.*
