# Supplementary material for: Open-Label Placebo Injection for Chronic Back Pain With Functional Neuroimaging: A Randomized Clinical Trial
Source: JAMA Netw Open. 2024 Sep 11;7(9):e2432427. doi: 10.1001/jamanetworkopen.2024.32427 (PMC11391328; doi:10.1001/jamanetworkopen.2024.32427)
Supplement: Supplement 3. — Data Sharing Statement [file jamanetwopen-e2432427-s003.pdf]

Data Sharing Statement

Ashar. Open-Label Placebo Injection for Chronic Back Pain. *JAMA Netw Open*. Published September 11, 2024. doi:10.1001/jamanetworkopen.2024.32427

Data
Data available: Yes
Data types: Deidentified participant data, Data dictionary
How to access data: Deidentified participant data and a data dictionary will be publicly posted on a data sharing site (e.g., figshare) by date of publication
When available: With publication

Supporting Documents
Document types: None

Additional Information
Who can access the data: To everyone: Deidentified participant data and a data dictionary will be publicly posted on a data sharing site (e.g., figshare) by date of publication
Types of analyses: Any purpose
Mechanisms of data availability: publicly posted
